# An epithelial-mesenchymal transition-related gene signature predicts prognosis, immune infiltration, and drug sensitivity in colorectal cancer

**DOI:** 10.20517/cdr.2026.37

**Published:** 2026-07-27

**Authors:** Xiaonan Shen, Hao Zhong, Wenqing Jia, Zichao Guo, Haiqin Song, Zhenghao Cai, Xi Cheng, Jianwen Li, Bo Feng, Haoran Feng, Fei Yue

**Affiliations:** ^1^Department of Gastroenterology, Ruijin Hospital, Shanghai Jiao Tong University School of Medicine, Shanghai 200025, China.; ^2^Department of General Surgery, Ruijin Hospital, Shanghai Jiao Tong University School of Medicine, Shanghai 200025, China.; ^#^Authors contributed equally.

**Keywords:** Epithelial-mesenchymal transition, tumor microenvironment, gene signature, colorectal cancer, drug resistance

## Abstract

**Aim:** Colorectal cancer (CRC) ranks among the most prevalent malignancies across the globe, with treatment resistance often closely linked to the complexity of the tumor microenvironment (TME). This study aims to establish a gene signature associated with epithelial-mesenchymal transition (EMT) that integrates TME dynamics, prognosis prediction, and drug resistance assessment in CRC.

**Methods:** We employed the Cancer Genome Atlas (TCGA) resource and bulk RNA-sequencing profiles linked to EMT to identify common differentially expressed genes (DEGs). An eight-gene signature was constructed using multivariable Cox regression analysis. The correlations of risk groups (scores) with overall survival, biological characteristics, and drug sensitivity were analyzed in CRC patients.

**Results:** Patients in the high-risk group exhibited significantly worse clinical outcomes than those in the low-risk group. Moreover, a lower risk score was significantly correlated with increased responsiveness to both immune checkpoint inhibitors and 5-fluorouracil in CRC patients. Additionally, experiments *in vitro* and *in vivo* confirmed that FABP4 functions as an oncogene in CRC by facilitating cell growth, migration, and chemotherapy resistance.

**Conclusion:** The EMT-associated gene signature holds significant value for predicting clinical prognosis, immunotherapy responsiveness, and chemotherapy sensitivity in CRC.

## INTRODUCTION

Colorectal cancer (CRC) remains one of the most common malignant cancers, with both its incidence and mortality steadily increasing in recent years^[1]^. Despite significant progress in multimodal therapies, including surgery, chemotherapy, and immunotherapy, the prognosis for advanced CRC patients continues to be unfavorable, with early lymph node metastasis and acquired chemoresistance constituting major clinical obstacles. Hence, identifying the mechanisms underlying chemoresistance in CRC and discovering novel therapeutic targets hold significant promise for improving therapeutic efficacy^[2]^.

Epithelial-mesenchymal transition (EMT) is a highly conserved biological program through which epithelial cells systematically dismantle their characteristic features - such as apical-basal polarity, tight junctions, and basement membrane adherence - and progressively acquire mesenchymal traits, including a spindle-like morphology, increased motility, and enhanced invasive capacity^[3]^. This transdifferentiation program is orchestrated by a network of transcription factors, including Snail, Zeb, and Twist families. During embryonic development, EMT is essential for gastrulation, neural crest formation, and organogenesis. In adult organisms, the process is mostly quiescent but can be reinitiated during tissue repair, wound healing, and fibrosis under pathological conditions. Critically, aberrant EMT reactivation in cancer constitutes a major driver of tumor initiation, metastatic progression, and therapeutic resistance. Moreover, EMT endows cancer cells with stem cell-like properties and confers resistance to both conventional chemotherapy and targeted therapies, thereby promoting cellular dormancy and drug tolerance. Through these mechanisms, EMT contributes to the establishment of minimal residual disease - a pool of surviving, quiescent tumor cells that persist after initial treatment. These dormant cells can later re-enter the cell cycle, ultimately seeding tumor recurrence and metastatic outgrowth^[4]^. Accumulating evidence from preclinical and early clinical studies underscores the critical role of EMT in CRC progression, metastasis, and drug resistance, highlighting its markers as promising prognostic indicators and therapeutic targets.

The tumor microenvironment (TME) is a highly heterogeneous and dynamic ecosystem composed of cancer cells, infiltrating immune cells (such as tumor-associated macrophages, T lymphocytes, and myeloid-derived suppressor cells), cancer-associated fibroblasts, endothelial cells, pericytes, and non-cellular components, including the extracellular matrix, cytokines, chemokines, growth factors, and metabolic byproducts^[5,6]^. The intricate cross-talk among these constituents forms a signaling network that profoundly influences tumor behavior. Importantly, the TME is not a passive scaffold but rather an active participant that continuously instructs cancer cells to adapt to changing conditions, including hypoxia, acidosis, mechanical stress, and immune pressure. These microenvironmental cues are now widely recognized as major drivers of cancer progression, metastasis, and therapeutic resistance. A critical event triggered by TME signaling is the induction of EMT, a process through which epithelial cells undergo a phenotypic switch to acquire mesenchymal characteristics. This transition enhances cellular motility and invasiveness, ultimately driving metastatic dissemination and cancer progression^[7,8]^. Notably, previous studies have established a close link between EMT and both cancer stem-like properties and drug resistance^[9,10]^. Activation of EMT can drive tumor cells toward a more invasive phenotype while simultaneously diminishing their chemosensitivity through remodeling of the surrounding TME. Cells occupying a partial or hybrid mesenchymal state - often termed quasi-mesenchymal - display markedly enhanced tolerance to standard anticancer treatments, including cytotoxic chemotherapy. This resistance arises from multifaceted adaptations, such as entry into quiescence, upregulation of anti-apoptotic proteins, and increased drug efflux capacity^[11-13]^. Although considerable progress has been made in elucidating EMT in CRC, many aspects of its biological functions and underlying mechanisms remain incompletely defined. In particular, how EMT precisely orchestrates TME components, and how TME signals in turn modulate EMT plasticity, are still not fully understood. Elucidating these reciprocal interactions holds promise for overcoming drug resistance and improving therapeutic outcomes.

This research employed the Cancer Genome Atlas (TCGA) database and bulk RNA-sequencing information associated with EMT to identify common differentially expressed genes (DEGs). Based on EMT-associated DEGs, an eight-gene signature was established by using multivariable Cox regression analysis. To comprehensively elucidate the heterogeneity of CRC, we established an EMT-related signature, which was subsequently used to compute a corresponding score for each individual sample. Strikingly, the EMT-related gene signature possessed robust potential to predict both clinicopathological features and survival outcomes in CRC patients. Furthermore, results indicated that the EMT-related gene signature could predict biological characteristics, immune responses, and drug resistance in CRC samples. To further explore its clinical utility, we evaluated the potential of the signature genes as predictive biomarkers for chemotherapy and immunotherapy response. Subsequently, the expression of signature genes was validated. Furthermore, to investigate the biological function of FABP4, experiments *in vitro* and *in vivo* were performed. Relevant results demonstrated that FABP4 knockdown suppressed the growth of CRC cells and increased sensitivity to oxaliplatin, indicating that FABP4 could act as a new treatment target for CRC. In summary, this systematic analysis elucidates the clinicopathological relevance, prognostic value, and drug sensitivity prediction of an EMT-related gene signature in CRC, while also uncovering its underlying molecular mechanisms. These insights open new avenues for identifying potential therapeutic targets.

## METHODS

### Data collection and preprocessing

Gene expression profiles and matching clinical details for both CRC and normal tissue samples were acquired from the Gene Expression Omnibus (GEO) and TCGA databases. This study incorporated three reliable CRC cohorts - TCGA-COADREAD, GSE39582, and GSE103479. Microarray data from GEO were normalized using the RMA algorithm based on corresponding platform annotations. RNA-sequencing results and clinical records for TCGA samples were downloaded from the TCGA data portal. Bulk RNA-seq data related to EMT and corresponding clinical information were ﻿collected from publicly available datasets in the Clinical Proteomic Tumor Analysis Consortium (CPTAC) colon cancer database^[14]^.

### Development and validation of an EMT-related gene signature

First, DEGs were screened between CRC and normal tissues using the TCGA dataset. Subsequently, a separate screening was performed to identify DEGs between EMT samples and non-EMT samples in an independent database. When the DEGs in the two datasets overlapped, a total of 289 genes were obtained (|log_2_FoldChange| > 1, *P* < 0.05). Univariate Cox regression analysis was then applied to these genes using the “survival” package in R software. The 35 genes with prognostic value were further included in a multivariate Cox regression framework to construct a risk scoring system. Each sample’s risk value was derived according to the equation: Risk score = h_0_(t) × exp(β_1_X_1_ + β_2_X_2_ + … + β_n_X_n_), in which n stands for the number of model genes, β is the regression coefficient, X represents the gene expression level, and h_0_(t) was produced by the “predict” tool. Based on the optimal threshold, patients in both training and validation sets were divided into high-risk and low-risk subgroups. Kaplan-Meier survival analysis was then used to compare survival outcomes between the two groups and assess the predictive performance of this eight-gene signature associated with EMT.

### Biological function analysis in high- and low-risk groups

To explore the functional differences between the high-risk and low-risk subgroups in the TCGA cohort, we carried out gene expression difference analysis with the R package “DESeq2”. DEGs were identified using |log_2_FoldChange| > 1 and *P* < 0.05. For Gene Ontology (GO) and Kyoto Encyclopedia of Genes and Genomes (KEGG) enrichment analysis of these DEGs, significantly enriched terms were determined using an FDR-adjusted *P*-value < 0.05, and the analysis was performed with the “clusterProfiler” R package. The enrichment results are presented as bar plots. In addition, Gene Set Variation Analysis (GSVA) analysis was performed using the R package “GSVA”, based on the reference gene set “c2.cp.kegg.v7.5.1.symbols.gmt” (downloaded from MSigDB), to elucidate the biological process differences between the two groups.

### Construction of nomogram for survival prediction

Risk scores derived from CRC samples were integrated with clinicopathological data (age, gender, pathological stage, and AJCC-TNM stage) for subsequent analysis. Univariate and multivariate Cox regression models were used to screen for independent prognostic factors with a statistical threshold of *P* < 0.05, and these significant factors were employed to construct a nomogram for individualized survival prediction. The predictive efficacy of the established nomogram was validated through 1-, 3- and 5-year receiver operating characteristic (ROC) curve analysis and calibration plots, which were performed with the R software packages of “survivalROC” and “rms”.

### Exploring the role of EMT-related gene signature in drug treatment

The drug sensitivity of each sample was predicted using the “pRRophetic” R package. This package utilizes gene expression data and corresponding drug sensitivity (IC_50_) information from the Cancer Genome Project (CGP) as the training set to build predictive models. To assess immunotherapeutic response, the tumor immune dysfunction and exclusion (TIDE) algorithm was employed (http://tide.dfci.harvard.edu/). Moreover, the external IMvigor210 dataset was utilized to further verify the predictive performance of the EMT-associated prognostic model in a separate immunotherapy patient cohort.

### Specimens and cell lines

This study was approved by the Biomedical Ethics Committee of Ruijin Hospital, Shanghai Jiao Tong University School of Medicine (No. 2021-28), with written informed consent obtained from all enrolled CRC patients. Human CRC cell lines HT29 and SW480, together with the normal colonic epithelial cell line NCM460, were purchased from Shanghai Shenger Biotechnology Co., Ltd., which originally sourced these cells from the American Type Culture Collection (ATCC). All cell lines were authenticated by STR profiling, performed by Suzhou Jianda Biotechnology Co., Ltd. in 2026. Routine mycoplasma testing was performed using a polymerase chain reaction (PCR)-based assay throughout the experimental period, and all lines were confirmed negative. Cells were used between passages 5 and 15 after thawing. All cell lines were cultured in RPMI-1640 medium supplemented with 10% fetal bovine serum (FBS) at 37 °C in a humidified atmosphere with 5% CO_2_. The RRIDs for HT29, SW480, and NCM460 are CVCL_0320, CVCL_0546, and CVCL_0460, respectively. Oxaliplatin resistant HT29 and SW480 cells were established by exposing their respective parental cells to stepwise increasing concentrations of oxaliplatin, with the final induction concentration reaching 2 µM over a period of 6 months. The established resistant sublines were subsequently maintained in RPMI 1640 medium supplemented with 10% FBS and 2 µM oxaliplatin to preserve the resistant phenotype. Resistance was validated by assessing the half-maximal inhibitory concentration using a CCK-8 assay, with the detailed IC_50_ values in Supplementary Table 1.

### Lentiviral vector construction and validation

Two independent short hairpin RNA (shRNA) sequences targeting human FABP4 (shFABP4-1: ACATGATCATCAGTGTGAATG; shFABP4-2: CGAGAGTTTATGAGAGAGCAT) and a non-targeting scramble control (shNC: TTCTCCGAACGTGTCACGT) were individually cloned into the lentiviral backbone PGMLV-hU6-H-shRNA-CMV-ZsGreen1-PGK-Puro (Shanghai Genomeditech Co., Ltd., China), which co-expresses a ZsGreen1 reporter and a puromycin resistance gene for fluorescent tracking and antibiotic selection. Lentiviral transduction was performed on CRC cells to establish stable cell lines. CRC cells were seeded 24 h prior to infection to reach 50%-70% confluence on the day of transduction. Lentiviral particles were added at a multiplicity of infection (MOI) of 30 in the presence of 8 µg/mL polybrene to enhance transduction efficiency. The virus-polybrene mixture was applied to cells and incubated at 37 °C for 24 h. The medium was then replaced with fresh complete medium, and cells were further cultured for 48 h to allow transgene expression. For stable cell line generation, transduced cells were selected with 2 µg/mL puromycin. The knockdown efficiency of FABP4 was then validated via Western blotting.

### Real-time quantitative polymerase chain reaction

Total RNA was extracted from cultured cells using the FastPure® RNA Isolation Kit V2 (Vazyme, China), and RNA concentration was determined by spectrophotometry. Reverse transcription was performed using HiScript® RT SuperMix with genomic DNA elimination reagents, with 1 µg of total RNA used per 20 µL reaction under the following conditions: 37 °C for 15 min, followed by 85 °C for 5 s. Quantitative real-time PCR was subsequently carried out using ChamQ Universal SYBR qPCR Master Mix following the manufacturer’s instructions. The thermal cycling protocol consisted of an initial denaturation at 95 °C for 30 s, followed by 40 cycles of 95 °C for 10 s and 60 °C for 30 s, with a final melt curve analysis to verify product specificity. All reactions were performed in technical triplicate. The primer sequences used in this study are listed in Supplementary Table 2.

### Western blot

CRC cells were lysed in RIPA buffer supplemented with protease and phosphatase inhibitor cocktails to preserve protein integrity. Protein concentrations were determined using a standard BCA protein assay kit, and equal amounts of total protein (20 µg per lane) were separated by SDS-PAGE and subsequently transferred onto PVDF membranes. The membranes were blocked with 5% non-fat milk in TBST for 1 h at room temperature to prevent nonspecific binding, followed by overnight incubation at 4 °C with primary antibodies against CD44, OCT4, SOX2, FABP4, and GAPDH (as the internal control). After thorough washing, the membranes were incubated with corresponding HRP-conjugated secondary antibodies (1:5,000) for 1 h at room temperature. Specific protein bands with immune reactivity were visualized using chemiluminescence imaging systems, and their gray values were quantitatively analyzed with ImageJ. Details of all antibodies are listed in the Supplementary Table 3.

### Cell viability assay

Cell proliferative capacity was assessed using the commercial CCK-8 detection method. CRC cells were seeded into 96-well culture plates at a density of 5 × 10^3^ cells per well, with each well supplemented with 100 μL of RPMI-1640 culture medium. At preset experimental time points, the original culture medium was replaced with fresh medium mixed with 10% CCK-8 working solution, and cells were incubated for two hours at 37 °C under dark conditions. A microplate reader was finally used to detect the optical density value at the wavelength of 450 nm.

### Wound healing and transwell migration assays

For the wound healing test, CRC cells were grown to full confluence in 6-well plates. A sterile pipette tip was used to create uniform scratches on the cell monolayer, followed by gentle PBS washing. The processed cells were then cultured in serum-free medium for a 48-hour incubation period. In the transwell migration experiment, 5 × 10^4^ suspended cells in serum-free medium were added to the upper inserts, while the lower compartments were filled with medium containing 20% FBS to induce cell migration. After incubation, migrated cells were fixed with 4% paraformaldehyde for 30 min and stained with 0.25% crystal violet solution for 25 min for subsequent observation and analysis.

### Animal study

Six-week-old male BALB/c nude mice were purchased from Phenotek Biotechnology (Shanghai) Co., Ltd. All animal experiments were approved by the Institutional Animal Care and Use Committee of Phenotek Biotechnology under protocol number (AUP-202511-02). All animal housing and experiments were conducted in strict accordance with the institutional guidelines for the care and use of laboratory animals. For subcutaneous xenograft establishment, HT29 cells (5 × 10^5^ cells per mouse in 100 µL PBS) were inoculated into the right lateral flank of each mouse. A total of 32 mice were used in this study, with 8 mice randomly assigned to each of the four experimental groups. Sample size for the primary endpoint (tumor growth) was determined by power analysis (α = 0.05, 1 - β = 0.80) based on preliminary data, indicating a minimum of 5 mice per group for the longitudinal tumor-growth cohort. An additional 3 mice per group were separately allocated as an independent cohort for histological and molecular analyses, yielding a total of 8 mice per group. In each group, five mice were designated for longitudinal tumor-volume monitoring (tumor-growth cohort). The remaining three mice per group were euthanized at predetermined intermediate time points for histological evaluation and molecular assays (tissue-analysis cohort). The two cohorts were independent, with no overlap. When tumor volumes reached approximately 100 mm^3^ [measured by digital caliper and calculated as volume = (length × width^2^)/2], mice were randomly allocated to treatment groups using a computer-generated randomization sequence. The investigator responsible for tumor measurements and data analysis was blinded to group allocation. Mice received intraperitoneal injections of either normal saline (control) or oxaliplatin (5 mg/kg) twice per week. Tumor size and body weight were recorded every two days throughout the treatment cycle. Each tumor was measured three times as technical replicates, with five mice per group as biological replicates for tumor growth analysis. Mice were monitored daily for signs of distress or morbidity. Humane endpoints included tumor volume exceeding 2,000 mm^3^, body weight loss > 20% of initial weight, tumor ulceration/necrosis, or moribund status; any animal reaching these endpoints was euthanized immediately. At the end of the experiment, the remaining five mice per group in the tumor-growth cohort were euthanized by cervical dislocation under isoflurane anesthesia. No animals met the exclusion criteria, and no animals were excluded from the final analysis or died unexpectedly during the experiment.

### Statistical analysis

All statistical analyses and data processing were performed using R software (version 4.1.2). All *in vitro* experiments were performed with at least three biological replicates, each containing three technical replicates. Unless otherwise indicated, data are presented as mean ± standard deviation (SD). Comparisons between two groups were assessed using unpaired two-tailed Student’s *t*-test, while comparisons among three or more groups were evaluated using one-way analysis of variance (ANOVA) followed by Tukey’s post-hoc test for multiple comparisons. For tumor growth curves, two-way repeated-measures ANOVA with Bonferroni post-hoc correction was applied. Patient survival profiles were analyzed using Kaplan-Meier curves. The “timeROC” package was used to generate time-dependent ROC curves to evaluate the predictive efficiency of the prognostic model, and univariate and multivariate Cox regression analyses were utilized to screen independent prognostic factors. A two-tailed *P* < 0.05 was considered statistically significant. For all figures, ns indicates *P* ≥ 0.05, ^*^P < 0.05, ^**^P < 0.01, and ^***^P < 0.001.

## RESULTS

### Identification of EMT-related DEGs associated with prognosis and ﻿construction of risk score

**Figure 1:**
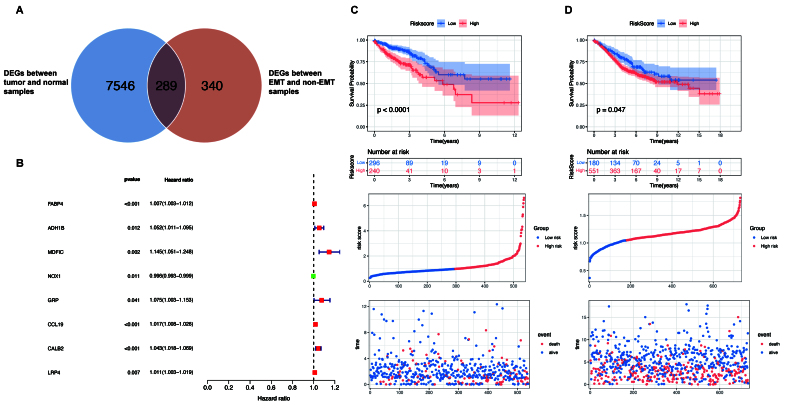
Construction and validation of EMT-related prognostic prediction model. (A) Identification of common DEGs across two datasets; (B) Development of an eight-gene prognostic signature via multivariate Cox regression analysis; (C and D) Survival comparison between low-risk and high-risk groups in the training and validation cohorts. Survival differences between groups were compared using the two-sided log-rank test. EMT: Epithelial-mesenchymal transition; DEGs: differentially expressed genes.

First, DEGs between CRC samples and normal tissues in the TCGA database were screened. In addition, DEGs between EMT samples and non-EMT samples from another database were selected. When the DEGs in the two datasets overlapped, a total of 289 genes were obtained [Figure 1A]. Thirty-five DEGs with prognostic value were preliminarily screened via univariate Cox regression analysis with a statistical threshold of *P* < 0.05, which were reserved for subsequent model building. An eight-gene signature was constructed based on multivariable Cox regression analysis [Figure 1B]. Hazard ratio (HR) values were computed for each candidate gene to assess its prognostic implications, with HR values less than 1 indicating protective effects and HR values greater than 1 suggesting adverse risk factors for CRC patients. A personalized scoring system termed the risk score was established based on the expression levels and corresponding regression coefficients of signature genes. This formula was applied to compute the individual risk score for every enrolled CRC specimen. Subsequent analyses were carried out to explore survival disparities among samples with varied risk score levels. Within the training set, a high-risk score was associated with poorer overall survival (OS) among CRC samples relative to a low-risk score [Figure 1C]. The validation sets further confirmed these observations [Figure 1D]. Collectively, these results demonstrated that the prognostic model constructed from EMT-associated genes could reliably predict survival status for CRC patients.

### Differences in biological features and TME immune infiltration across risk subgroups

**Figure 2:**
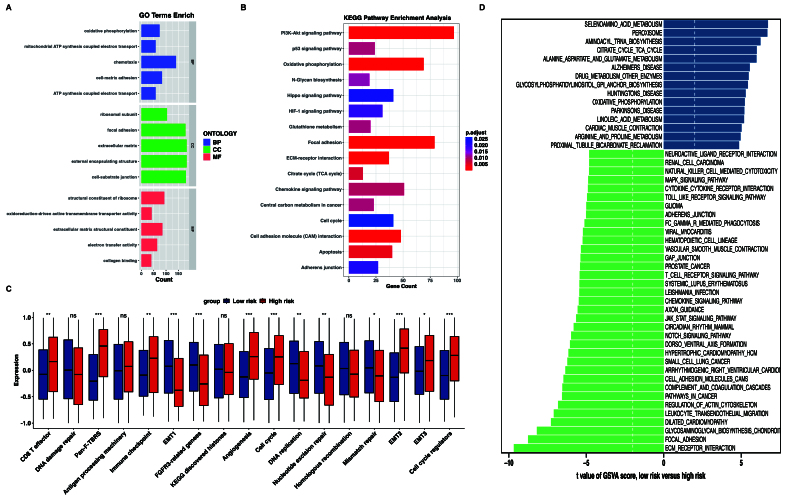
Biological and TME infiltration characteristics in the high- and low-risk groups. (A) Functional enrichment of DEGs via GO analysis comparing the high-risk and low-risk subgroups; (B) KEGG pathway enrichment of DEGs derived from the two groups; (C) GSVA was utilized to quantify and compare varied biological processes across different risk subgroups; (D) Bar charts illustrating the GSVA scores of typical KEGG signaling pathways collected from MSigDB in distinct groups. ns indicates *P* ≥ 0.05, * indicates *P* < 0.05, ** indicates *P* < 0.01, and *** indicates *P* < 0.001. TME: Tumor microenvironment; DEGs: differentially expressed genes; GO: Gene Ontology; KEGG: Kyoto Encyclopedia of Genes and Genomes; GSVA: Gene Set Variation Analysis; ATP: adenosine triphosphate; BP: biological process; CC: cellular component; MF: molecular function; ECM: extracellular matrix; TCA: tricarboxylic acid cycle.

To further explore the underlying molecular mechanisms, GO enrichment analysis of DEGs between two groups was conducted. Results revealed top-ranked pathways, including chemotaxis, cell-matrix adhesion, focal adhesion, and extracellular matrix structural constituent [Figure 2A]. Furthermore, KEGG pathway analysis underscored a notable enrichment in the extracellular matrix (ECM)-receptor interaction, Hippo signaling, Cell cycle, and Apoptosis pathways [Figure 2B]. GSVA was then applied to dissect discrepant biological functions across the two groups. The high-risk group displayed enhanced stromal-related biological activities, such as EMT2/3 and angiogenesis [Figure 2C]. Meanwhile, GSVA results also indicated prominent enrichment of stroma-associated signaling events such as ECM-receptor interaction and focal adhesion in the high-risk group [Figure 2D]. In summary, the findings above suggested that CRC patients with different risk scores exhibited differences in prognosis, biological activity, and TME, providing new insights into CRC research.

### Construction and validation of a prognostic nomogram

**Figure 3:**
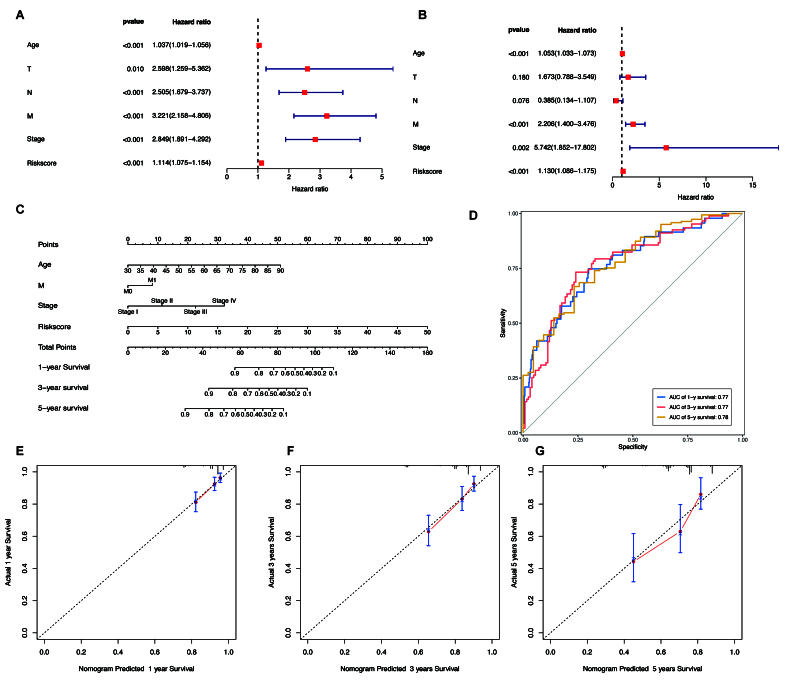
Construction of a prognostic nomogram for CRC patients. (A and B) Forest plot visualizing the univariate and multivariate Cox regression outcomes of the risk score and diverse clinical features; (C) The integrated nomogram model established based on four independent prognostic factors: age, pathological stage, M stage and individualized risk score; (D) Time-dependent ROC curves assessing the predictive efficiency for 1-year, 3-year and 5-year survival status; (E-G) Calibration curves of the nomogram for survival prediction at the three key time points (1-, 3- and 5-year). CRC: Colorectal cancer; ROC: receiver operating characteristic; AUC: area under the curve.

Independent prognostic factors - including age, pathological stage, M stage, and risk score - were identified via univariate and multivariate Cox regression [Figure 3A and B]. A comprehensive prognostic nomogram integrating these variables was constructed to estimate 1-, 3-, and 5-year OS in CRC patients [Figure 3C]. Time-dependent ROC curve analysis was conducted to assess model performance, with AUC values calculated as 0.77, 0.77, and 0.78 at the three corresponding time points, thereby validating the stable and powerful predictive efficacy of this model [Figure 3D]. Meanwhile, calibration curve results showed great consistency between survival outcomes predicted by the nomogram and actual clinical observations [Figure 3E-G], indicating that this nomogram can serve as a reliable auxiliary tool for precise prognostic stratification of CRC cases.

### Correlation of the risk score with the tumor immune microenvironment and drug sensitivity

**Figure 4:**
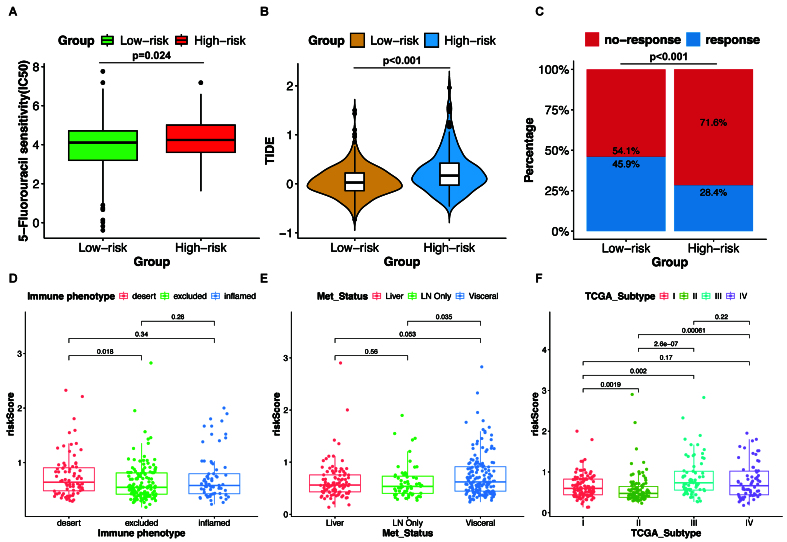
Analysis of tumor immune microenvironment features and drug susceptibility across distinct risk subgroups. (A) Differential therapeutic responses to 5-FU treatment between high-risk and low-risk groups; (B) Quantitative comparison of TIDE scores between the two risk subgroups; (C) The fraction of patients with immunotherapy response in two groups; (D-F) Associations of the risk score with the immune phenotype, metastatic state, and TCGA subtype, respectively. For (A, B, D-F), statistical significance was assessed using two-sided Wilcoxon rank-sum tests. For (C), the difference in response proportions between the two groups was evaluated using Pearson’s Chi-square test. 5-FU: 5-Fluorouracil; TIDE: tumor immune dysfunction and exclusion; TCGA: the Cancer Genome Atlas.

Additional explorations were performed to clarify the correlation between calculated risk scores and drug susceptibility. For chemotherapeutic efficacy evaluation, high-risk samples presented notably higher IC_50_ levels of 5-fluorouracil than low-risk counterparts (*P* = 0.024; Figure 4A), suggesting that CRC patients with elevated risk score may derive limited benefit from conventional chemotherapy. Considering the widespread clinical application and promising anti-tumor effects of immune checkpoint inhibitors (ICIs), the TIDE algorithm was adopted to explore whether this risk model could predict immunotherapeutic efficacy. Patients in the high-risk subgroup had markedly elevated TIDE scores relative to low-risk cases [Figure 4B]. In line with this result, low-risk individuals achieved a better immune treatment response rate, reaching 45.9% compared with 28.4% in the high-risk group [Figure 4C]. Moreover, Wilcoxon rank-sum tests confirmed significant correlations between risk score levels and core clinical characteristics in the IMvigor210 cohort, covering immune phenotype, metastatic status, and TCGA molecular subtype [Figure 4D-F]. All the above analytical results strongly suggested that the proposed risk signature is closely linked to both chemotherapeutic sensitivity and immunotherapeutic responsiveness in CRC.

### Validation of the expression and function of model-associated genes

**Figure 5:**
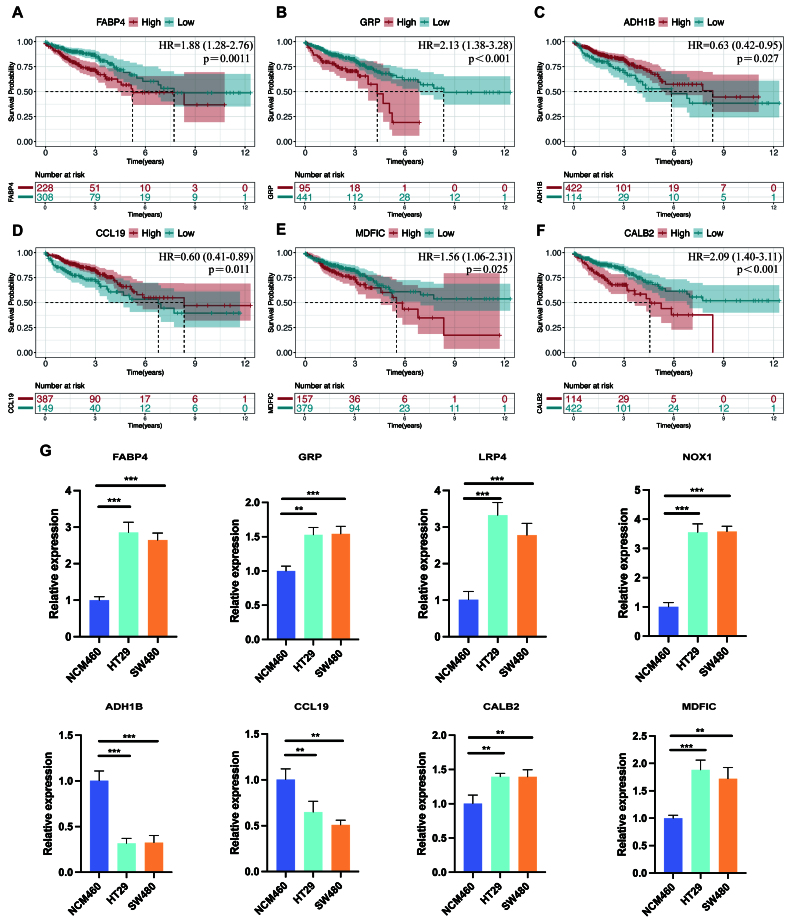
Validation of the expression and function of model-associated genes. (A-F) The expression of FABP4, GRP, ADH1B, CCL19, CALB2, and MDFIC was a significant prognostic indicator in CRC (log-rank test, two-sided); (G) The expression levels of model-associated genes in the normal cell line (NCM460) and CRC cell lines (HT-29, SW480) were detected by RT-qPCR. Data are presented as mean ± SD from three independent biological replicates. Statistical significance was determined by one-way ANOVA followed by Tukey’s post-hoc test for multiple comparisons. ** indicates *P* < 0.01, and *** indicates *P* < 0.001. CRC: Colorectal cancer; RT-qPCR: reverse transcription quantitative polymerase chain reaction; SD: standard deviation; ANOVA: analysis of variance; HR: hazard ratio.

Subsequently, the function of model-associated genes was evaluated. Among them, the expression of FABP4, GRP, ADH1B, CCL19, CALB2, and MDFIC were significant prognostic indicators [Figure 5A-F]. Specifically, elevated levels of FABP4, GRP, CALB2, and MDFIC were correlated with a poor prognosis in CRC. Conversely, reduced expression of ADH1B and CCL19 was also significantly associated with adverse patient outcomes. To verify the clinical and biological relevance of the established EMT-associated gene signature at the cellular level, we applied reverse transcription quantitative polymerase chain reaction (RT-qPCR) to detect the expression profiles of signature genes. Results showed that the expression levels of FABP4, GRP, LRP4, NOX1, CALB2, and MDFIC were up-regulated in CRC cells compared with NCM460. On the contrary, the expression levels of ADH1B and CCL19 were down-regulated [Figure 5G].

### FABP4 serves as a biomarker to predict prognosis and drug sensitivity in CRC

**Figure 6:**
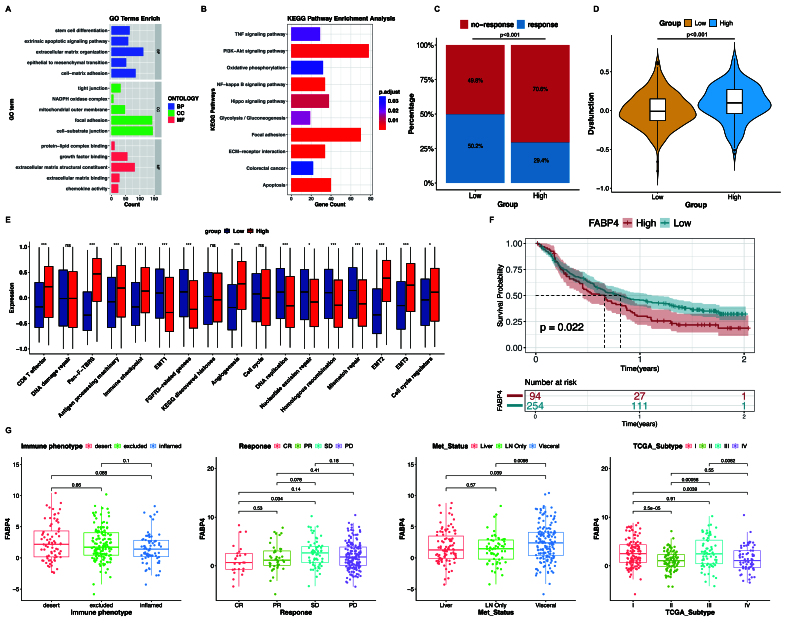
Correlation of biological signatures and immunotherapy response with FABP4 expression levels. (A) Functional GO enrichment of DEGs identified from FABP4-high and FABP4-low subgroups; (B) KEGG pathway enrichment based on DEGs screened from the two groups; (C) Variations in the proportion of responsive cases to immune treatment among different FABP4 expression subgroups; (D) The dysfunction score in the two groups was detected by TIDE analysis; (E) GSVA analysis was used to detect the differences in biological processes between the two groups; (F) Survival outcomes of the two patient groups in the IMvigor210 cohort; (G) The correlations between the level of FABP4 and immune phenotype, treatment response, metastatic state, and TCGA subtype. For (D and G), statistical significance was assessed using two-sided Wilcoxon rank-sum tests. For (C), the difference in response proportions between the two groups was evaluated using Pearson’s Chi-square test. * indicates *P* < 0.05, and *** indicates *P* < 0.001. GO: Gene Ontology; DEGs: differentially expressed genes; KEGG: Kyoto Encyclopedia of Genes and Genomes; TIDE: tumor immune dysfunction and exclusion; GSVA: Gene Set Variation Analysis; TCGA: the Cancer Genome Atlas; BP: biological process; CC: cellular component; MF: molecular function; CR: complete response; PR: partial response; SD: stable disease; PD: progressive disease.

Several studies have implicated FABP4 in promoting tumorigenesis and contributing to drug resistance in other cancers, such as ovarian and breast cancer^[15,16]^. To further explore the role of FABP4 in CRC, CRC samples were stratified into two groups based on the expression level of FABP4. GO enrichment analysis of DEGs between the two groups revealed top-ranked pathways including epithelial-to-mesenchymal transition, cell-matrix adhesion, tight junction, and extracellular matrix structural constituent [Figure 6A]. KEGG enrichment analysis identified Focal adhesion, Hippo signaling, and Apoptosis as significantly modulated pathways [Figure 6B]. Consistent with this, FABP4 was implicated in the regulation of critical oncogenic processes in CRC, including EMT and apoptosis. In the TIDE analysis, CRC samples with a high level of FABP4 were associated with tumor immune dysfunction, suggesting a lower therapeutic benefit and immune response to ICI treatment in these patients [Figure 6C and D]. We further examined the differential enrichment of known biological pathways between the two groups [Figure 6E]. Samples with a high level of FABP4 displayed elevated stromal functional activities, including activated EMT2/3 signaling and angiogenesis. In the IMvigor210 cohort, elevated FABP4 expression was correlated with adverse survival outcomes for tumor patients [Figure 6F]. In addition, there were significant associations between FABP4 levels and immune phenotype, treatment response, metastatic state, or TCGA subtype [Figure 6G]. To experimentally validate the bioinformatic predictions regarding the tumor immune microenvironment, immunofluorescence (IF) staining was performed on CRC tissue samples stratified by FABP4 expression levels. As shown in Supplementary Figure 1, tumors with high FABP4 expression exhibited significantly stronger α-SMA fluorescence intensity compared with their low-expression counterparts, indicating pronounced stromal activation and increased cancer-associated fibroblast infiltration. Furthermore, IF analysis of immune cell markers [Supplementary Figure 2] revealed that high-FABP4 tumors harbored a marked increase in CD8^+^ T-cell infiltration. Notably, this enhanced CD8^+^ infiltration was accompanied by concurrent upregulation of PD-L1 expression within the TME. Collectively, these findings demonstrate that FABP4 is closely associated with stromal remodeling and the establishment of an immunosuppressive PD-L1/PD-1 axis, which may have important implications for the efficacy of clinical immunotherapy in CRC.

### FABP4 was upregulated in CRC, and its biological behavior was verified

**Figure 7:**
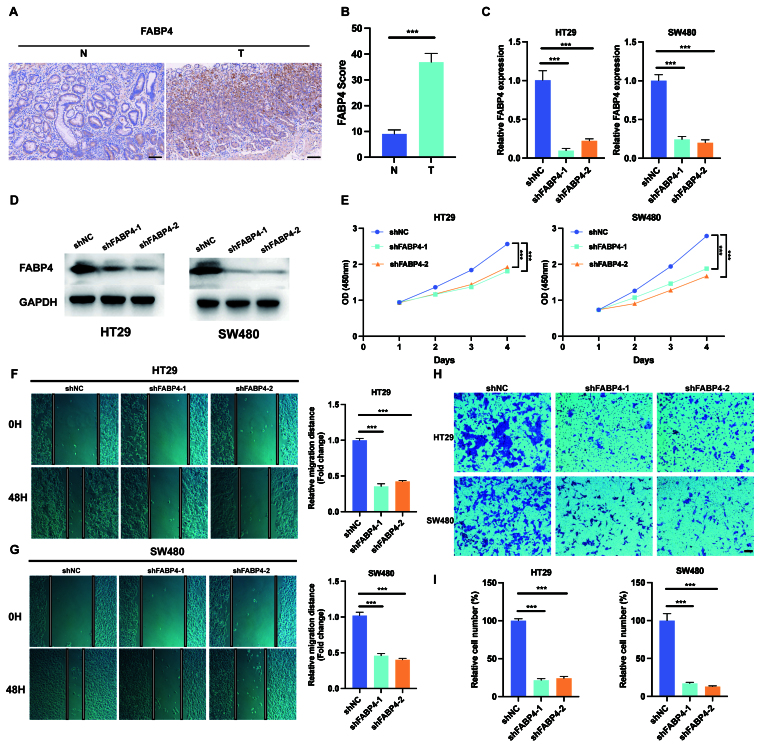
The role of FABP4 in promoting CRC was validated *in vitro*. (A and B) Tissue samples collected from CRC patients were examined to assess the expression status of FABP4 (Scale bar = 0.1 mm); (C and D) The FABP4 knockdown efficiency was validated by RT-qPCR and western blotting; (E) CCK-8 assay showed that FABP4 knockdown suppressed CRC growth; (F and G) FABP4 knockdown attenuated tumor cell migration in wound healing assays; (H and I) Transwell assay indicated that FABP4 knockdown suppressed tumor cell migration ability (Scale bar = 0.2 mm). Data are presented as mean ± SD from three independent biological replicates. Comparisons between two groups were performed using unpaired Student’s *t*-test, whereas comparisons among three groups were performed using one-way ANOVA followed by Tukey’s post-hoc test. *** indicates *P* < 0.001. CRC: Colorectal cancer; RT-qPCR: reverse transcription quantitative polymerase chain reaction; SD: standard deviation; ANOVA: analysis of variance.

To assess the function of FABP4 in CRC, experiments *in vitro* were performed. Immunohistochemical analysis of clinical specimens confirmed that FABP4 was upregulated in tumor lesions relative to paired peri-tumor normal tissues [Figure 7A and B]. FABP4 knockdown in HT29 and SW480 cells was achieved via shRNA transduction, with efficiency confirmed by RT-qPCR and western blotting at the mRNA and protein levels, respectively [Figure 7C and D]. CCK-8 results illustrated that FABP4 knockdown greatly suppressed the proliferative ability of CRC cells [Figure 7E]. Additionally, both wound healing and transwell migration assays showed that suppression of FABP4 markedly weakened the migratory capacity of CRC cells [Figure 7F-I]. All these outcomes demonstrate that FABP4 exerts critical oncogenic effects by facilitating CRC cell growth and migration.

### FABP4 promoted resistance to oxaliplatin in CRC

**Figure 8:**
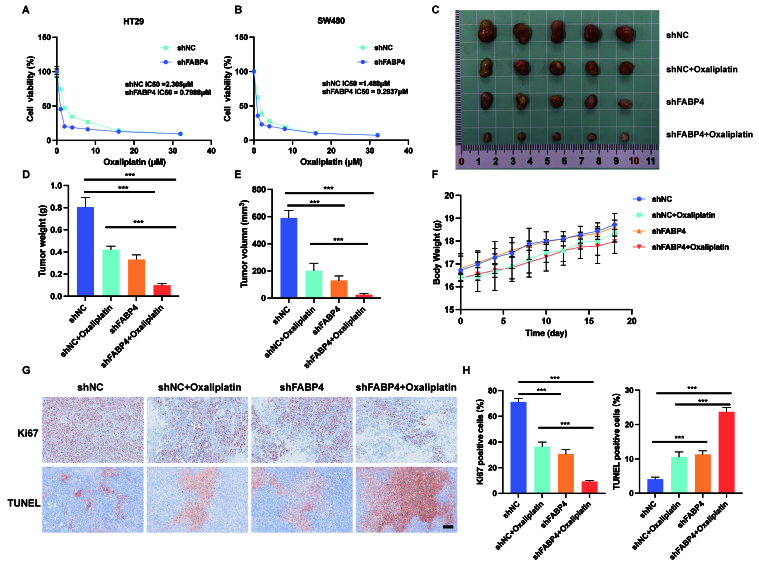
FABP4 promoted resistance to oxaliplatin in CRC. (A and B) Enhanced oxaliplatin susceptibility was observed in CRC cells after FABP4 knockdown (*n* = 3 biological replicates); (C) Representative images of tumors from four groups (shNC, shNC + oxaliplatin, shFABP4, shFABP4 + oxaliplatin) (*n* = 5 per group); (D) The weight of tumors from four groups; (E) The volume of tumors from four groups measured at the end of the experiment; (F) Body weight measurements of mice across the experimental period; (G) Ki67 and TUNEL staining images of tumors after different treatments (Scale bar = 0.2 mm; *n* = 3 per group); (H) The quantitative analysis of Ki67 and TUNEL staining. Data are presented as mean ± SD. Statistical significance was determined by one-way ANOVA followed by Tukey’s post-hoc test for multiple comparisons. *** indicates *P* < 0.001. CRC: Colorectal cancer; SD: standard deviation; ANOVA: analysis of variance.

To further investigate the effect of FABP4 on chemoresistance, CCK-8 assays were performed. Cellular assays showed that FABP4 knockdown markedly enhanced the sensitivity of CRC cells to oxaliplatin relative to shNC cells [Figure 8A and B]. Subsequent PCR and western blot detection consistently verified elevated FABP4 expression in chemotherapy-resistant CRC cell lines [Supplementary Figures 3 and 4]. Functional validation further confirmed that FABP4 knockdown effectively reversed the drug resistance phenotype and enhanced oxaliplatin responsiveness in resistant tumor cells [Supplementary Figure 5]. Mechanistic exploration via western blot assays illustrated that FABP4 downregulation suppressed the expression of stemness markers including CD44, OCT4, and SOX2, which further restored tumor cell sensitivity to oxaliplatin [Supplementary Figure 6]. To corroborate our *in vitro* findings, the effect of FABP4 knockdown on tumor growth and drug resistance was assessed in a subcutaneous xenograft model. Tumors derived from FABP4-depleted HT29 cells exhibited significantly reduced growth compared to controls. Importantly, the combined intervention of FABP4 knockdown and oxaliplatin administration produced the strongest anti-tumor efficacy, with the smallest tumor volume and weight observed among all experimental groups [Figure 8C-E, Supplementary Figure 7]. Body weight analysis revealed no significant differences among experimental groups [Figure 8F], indicating that FABP4 knockdown and oxaliplatin treatment were not associated with detectable toxicity *in vivo*. In addition, Ki67 and TUNEL staining of tumors from four groups were performed. IHC staining results showed a similar trend, with the most significant decrease in cell proliferation and increase in cell apoptosis observed in the combination of FABP4 knockdown and oxaliplatin treatment group [Figure 8G and H]. Taken together, these experimental results indicate that FABP4 suppression restrains CRC cell growth and elevates cellular responsiveness to oxaliplatin, implying its potential as an innovative therapeutic target for CRC clinical treatment.

## DISCUSSION

EMT is a fundamental biological reprogramming event. During development and tissue repair, it enables epithelial cells to adopt a mesenchymal phenotype. In cancer, however, this process is co-opted, conferring aggressive traits on tumor cells that drive disease progression. Upon EMT activation, tumor cells undergo characteristic changes - disruption of tight junctions, loss of apical–basal polarity, and extensive cytoskeletal reorganization - that collectively equip them to detach from the primary tumor, invade the surrounding stroma, survive in circulation, and ultimately establish metastases at distant sites^[17-20]^.

Numerous studies have validated that epithelial-mesenchymal transition exerts vital pro-migratory and pro-metastatic effects in multiple human malignancies, such as CRC, pancreatic cancer, liver cancer, and other cancers^[21-23]^. EMT is intricately linked to other key features of cancer, such as the acquisition of stem cell-like properties, metabolic reprogramming, and alterations within the TME^[24-26]^. The intricate interplay among these interconnected mechanisms produces a synergistic effect that collectively governs CRC progression and metastatic spread. Rather than functioning in isolation, these mechanisms converge to form a robust regulatory network that fuels tumor advancement and spread. This complexity yields a dual-edged reality for therapeutic development: the same pathways that sustain malignancy also present exploitable vulnerabilities. Consequently, overcoming treatment resistance demands strategies capable of disrupting this coordinated network, while the underlying interdependence opens avenues for combination therapies and precision interventions that could transform current clinical paradigms. Notably, EMT signatures are increasingly recognized as potential prognostic biomarkers, particularly in relation to circulating tumor cells and metastatic behavior^[27]^. Moreover, EMT is closely associated with therapeutic resistance in CRC, representing a major cause of subsequent treatment failure^[28,29]^. Hence, understanding how EMT-related gene signatures influence the TME and the molecular mechanisms involved may help reveal their roles in antitumor immunity and guide the development of more effective therapies.

This work analyzed transcriptomic profiles and clinical information from CRC patient datasets and screened out 35 prognosis-relevant DEGs linked to EMT. Using these EMT-associated molecular markers, we constructed an eight-gene predictive model to achieve more precise survival evaluation for CRC samples. The eight genes were FABP4, ADH1B, MDFIC, NOX1, GRP, CCL19, CALB2 and LRP4. RT-qPCR assays were carried out to verify the expression level of these screened target genes. Kaplan-Meier survival analysis revealed that patients with lower risk scores achieved remarkably longer survival time than those in the high-risk group. With high predictive consistency, discriminative capability, and accuracy, this signature effectively stratified patients into distinct prognostic groups. Subsequent univariate and multivariate Cox regression analyses further validated that this risk-based scoring system could serve as an independent prognostic factor for CRC. By integrating risk score with other significant clinical variables, we developed a prognostic nomogram to predict individual survival probabilities. The nomogram exhibited robust predictive performance, as demonstrated by time-dependent ROC curves and calibration plots, indicating high accuracy and reliability in prognostic estimation.

To better elucidate the role of the EMT-related model in predicting TME characteristics, we conducted GO, KEGG, and GSVA functional analyses on DEGs across distinct risk subgroups. The top-ranked pathways included cell-matrix adhesion, extracellular matrix structural constituent, Hippo signaling, and cell cycle. Additional functional evaluation also identified distinct biological behaviors between the two risk subgroups. The level of stromal activities such as EMT2/3 and angiogenesis was higher in samples with a high-risk score. Collectively, our results demonstrate that stratification by EMT score reveals significant differences in CRC patient prognosis, biological processes, and tumor immune infiltration. These collective findings indicate the reliability of this EMT scoring system for dissecting the intricate biological characteristics of CRC.

Given that chemotherapy remains the primary treatment modality for CRC, we attempted to explore the predictive value of the EMT-based model for chemotherapeutic susceptibility. Our findings revealed a significantly higher 5-FU IC_50_ in the high-risk group compared to the low-risk group, indicating that tumors with an elevated risk score are potentially refractory to conventional chemotherapy. Meanwhile, the emergence of immunotherapy has transformed the therapeutic landscape for various solid tumors, and its importance is well established in a distinct subset of CRC^[30,31]^. In particular, anti-PD-1 ICIs, including pembrolizumab and nivolumab, have exhibited prominent therapeutic advantages for metastatic CRC cases with dMMR and MSI-H molecular features^[32]^. Therefore, TIDE analysis, a newly identified predictor of immune response, was employed in this study. The results indicated that TIDE values were significantly increased in the high-risk group compared to their counterparts in the low-risk group. Patients in the low-risk group gained more clinical benefits and exhibited favorable immune reactions toward ICI-based immunotherapy. Overall, our results suggest that the risk score holds potential as an effective predictive biomarker for therapeutic response, showing clear associations with the efficacy of chemotherapy and immunotherapy.

FABP4, a member of the fatty acid-binding proteins (FABPs) family, modulates multiple core cellular activities such as metabolic reprogramming, signal transduction, and gene transcription. These activities enable FABP4 to integrate lipid signaling with broader cellular functions, influencing inflammation, endoplasmic reticulum stress, and cell proliferation. Accumulating evidence establishes that these regulatory mechanisms enable FABP4 to actively modulate both the initiation and progression of human malignancies^[33]^. Metabolic reprogramming, now recognized as a hallmark of cancer, reflects the inherent capacity of malignant cells to remodel their metabolic networks in response to intrinsic oncogenic signals and extrinsic environmental cues. To sustain proliferation under conditions of limited nutrient availability, cancer cells undergo adaptive metabolic alterations that enable sufficient energy production and the accumulation of biosynthetic precursors necessary for macromolecule synthesis. These adaptations not only support rapid cell division but also reinforce the malignant phenotype and facilitate aggressive tumor progression^[34]^. A well-characterized manifestation of this reprogramming is the shift toward aerobic glycolysis, widely known as the Warburg effect. This unique metabolic adaptation allows cancer cells to generate ATP and various metabolic intermediates even under normal oxygen conditions. Beyond glucose metabolism, alterations in lipid metabolism have emerged as equally critical components of the metabolic rewiring in cancer. Within this context, FABPs serve as key mediators that integrate lipid signaling with cellular metabolic demands. Among the FABP family, FABP4 has drawn substantial interest for its functional relevance in tumor biology. Mechanistically, FABPs support the elevated proliferative requirements of tumors by facilitating fatty acid uptake, promoting β-oxidation, and modulating lipid synthesis pathways. These coordinated activities provide cancer cells with both the energy and the structural components required for sustained growth. Moreover, FABP4 contributes to tumor progression through a diverse array of molecular mechanisms that extend beyond its conventional lipid transport function. Notably, it has been implicated in the rewiring of metabolic phenotypes, the epigenetic deregulation of DNA methylation patterns, and the activation of oncogenic signaling cascades^[35-37]^. In multiple malignant tumors, FABP4 promotes tumor transformation, proliferation, metastasis, and therapy resistance^[38-40]^. In the present study, we identified FABP4 as a tumor-promoting factor in CRC, where it enhances proliferative and migratory capacities and confers chemoresistance. Notably, elevated FABP4 levels correlate significantly with poor clinical outcomes, underscoring its prognostic relevance in CRC. Beyond its impact on tumor progression and prognosis, high FABP4 expression is also associated with diminished responsiveness to conventional chemotherapy and immunotherapy. These observations point to a broader role for FABP4 in shaping the therapeutic landscape of CRC, potentially influencing treatment efficacy through multiple modalities. The consistent association between FABP4 expression and resistance to diverse therapeutic strategies underscores its potential involvement in fundamental mechanisms of treatment failure, such as metabolic adaptation and evasion of immune surveillance. Given its multifaceted role in driving malignant behavior and modulating treatment sensitivity, FABP4 may represent a promising and previously underexplored therapeutic target in CRC. Targeting this molecule could offer a dual advantage - simultaneously restraining tumor aggressiveness while enhancing the efficacy of existing therapies. Collectively, these findings provide a compelling rationale for further investigation into FABP4 as a candidate for precision oncology strategies aimed at overcoming resistance and improving patient outcomes.

Collectively, our comprehensive bioinformatic analyses across multiple public datasets successfully constructed and verified a prognostic signature based on EMT-associated genes for CRC research. This novel scoring system can evaluate patient survival status, tumor immune infiltration patterns, and drug responsiveness. It provides reliable molecular evidence for predicting clinical responses to chemotherapy and immunotherapy in CRC cases. Furthermore, a series of experiments *in vitro* and *in vivo* were conducted to verify the biological function of FABP4. Results strongly demonstrated that FABP4 acts as an oncogene, markedly promoting the growth, migration, and chemotherapy resistance of CRC cells. These discoveries offer a new molecular target for the clinical treatment of CRC.

Nevertheless, our study has several limitations. First, the immunotherapy-related findings lack experimental validation. The immune landscape analyses are purely bioinformatic and have not been validated in animal models or clinical cohorts. Second, while we have demonstrated that FABP4 knockdown suppresses cancer stem cell properties, other potential mechanisms (e.g., drug efflux, apoptosis pathway modulation) remain to be explored. Third, the potential synergistic effects of targeting FABP4 in combination with immunotherapy have not been evaluated in animal models. Future studies should conduct experiments to validate the immunomodulatory role of FABP4 and evaluate combination strategies with immunotherapy. Fourth, experiments are needed to determine whether FABP4 regulates drug efflux pump activity and apoptosis signaling in CRC cells. Finally, the clinical feasibility of targeting FABP4 using small-molecule inhibitors or degraders in combination with standard chemotherapy warrants further exploration.

## DECLARATIONS

### Availability of data and materials

Additional information is available in the Supplementary Materials. RNA-seq and corresponding clinical data for the combined TCGA-COAD and TCGA-READ cohorts were retrieved from the TCGA program (https://www.cancer.gov/tcga). Two independent gene expression datasets, GSE39582 and GSE103479, were downloaded from the GEO database (https://www.ncbi.nlm.nih.gov/geo/). CPTAC data were obtained via the GDC Data Portal (https://portal.gdc.cancer.gov/). The IMvigor210 dataset was accessed via the IMvigor210CoreBiologies R package.

### AI and AI-assisted tools statement

During the preparation of this manuscript, the AI tool DeepSeek (version DeepSeek-V3.2, released 2025-12-1) was used solely for language editing. It did not influence the study design, data collection, analysis, interpretation, or the scientific content of the work. All authors take full responsibility for the accuracy, integrity, and final content of the manuscript.

### Copyright

© The Author(s) 2026.
