# Supplementary material for: An epithelial-mesenchymal transition-related gene signature predicts prognosis, immune infiltration, and drug sensitivity in colorectal cancer
Source: Cancer Drug Resist. 2026 Jul 27;9:25. doi: 10.20517/cdr.2026.37 (PMC13459117; doi:10.20517/cdr.2026.37)
Supplement: Supplementary file 1 [file cdr-9-25-SupplementaryMaterials.pdf]

Supplementary Materials

An epithelial-mesenchymal transition-related gene signature predicts prognosis, immune infiltration, and drug sensitivity in colorectal cancer

Xiaonan Shen[1,#], Hao Zhong[2,#], Wenqing Jia[2,#], Zichao Guo[2], Haiqin Song[2], Zhenghao Cai[2], Xi Cheng[2], Jianwen Li[2], Bo Feng[2], Haoran Feng[2], Fei Yue[2]

[1]Department of Gastroenterology, Ruijin Hospital, Shanghai Jiao Tong University School of Medicine, Shanghai 200025, China.
[2]Department of General Surgery, Ruijin Hospital, Shanghai Jiao Tong University School of Medicine, Shanghai 200025, China.
[#]Authors contributed equally.

Correspondence to: Dr. Jianwen Li, Dr. Bo Feng, Dr. Haoran Feng, Dr. Fei Yue, Department of General Surgery, Ruijin Hospital, Shanghai Jiao Tong University School of Medicine, Shanghai 200025, China. E-mail: ljw10499@rjh.com.cn; fb11427@rjh.com.cn; fhr01K33@rjh.com.cn; yuefei@shsmu.edu.cn

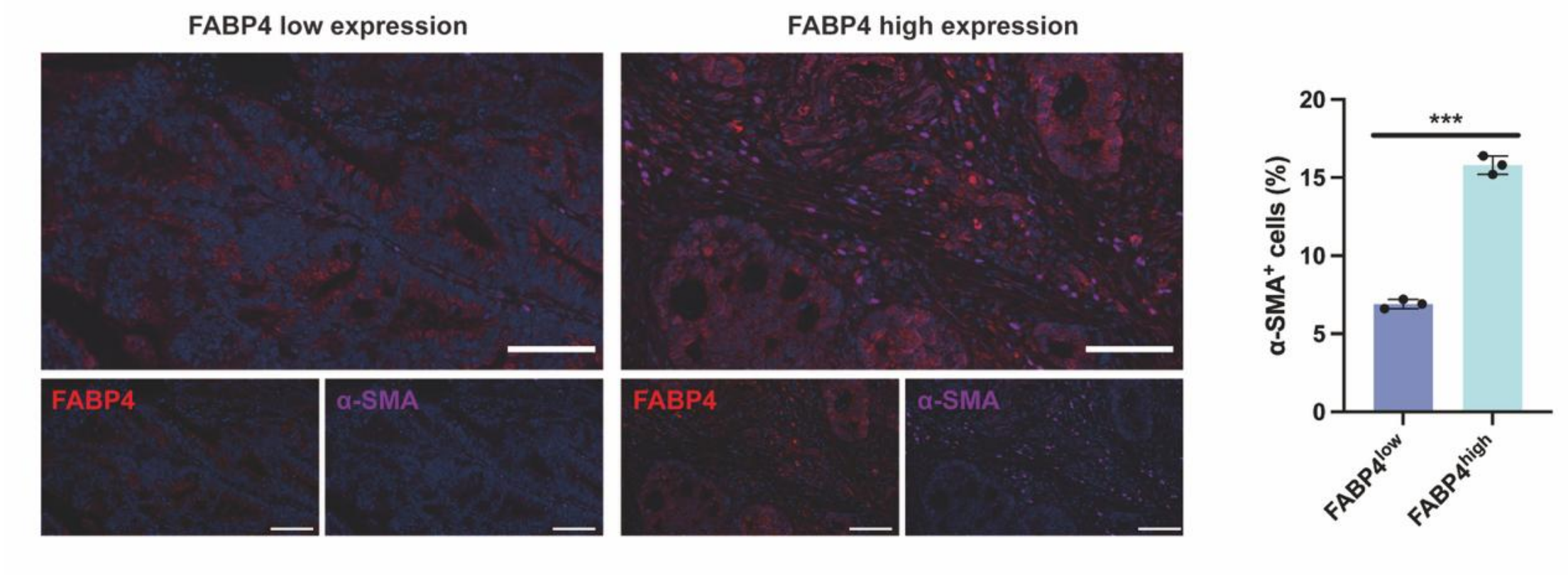

Supplementary Figure 1. Representative images of multiplex immunofluorescence in FABP4-low and FABP4-high human CRC tissues. Three representative patient samples per group were analyzed, with three sections per sample examined as technical replicates. Scale bar = 0.1 mm. Quantification of α-SMA^+ cells as a proportion of total cells. *p < 0.05, **p < 0.01, and ***p < 0.001.

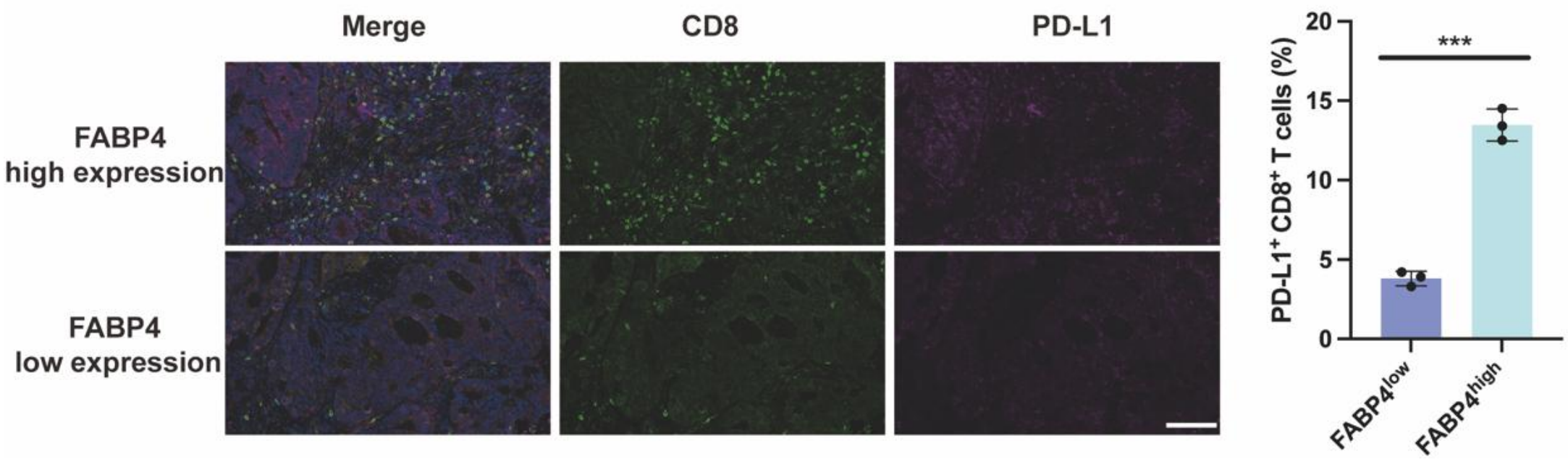

Supplementary Figure 2. The correlations between FABP4 expression and PD-$L1^+$ $CD8^+$ T cells were analyzed by immunofluorescence. Three representative patient samples per group were analyzed, with three sections per sample examined as technical replicates. Scale bar = 0.1 mm. *p < 0.05, **p < 0.01, and ***p < 0.001.

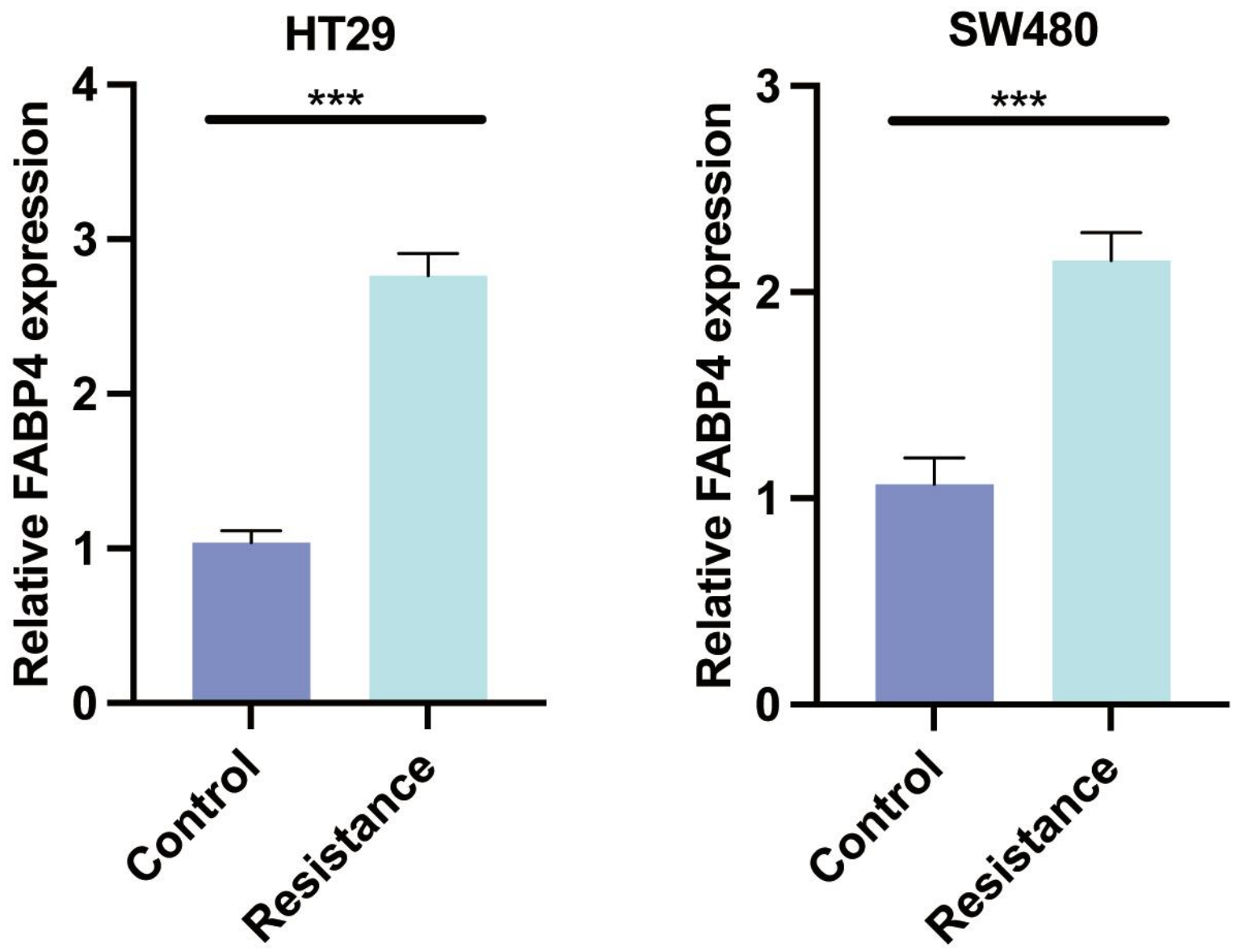

Supplementary Figure 3. The mRNA level of FABP4 in drug-resistant cell lines and control cells.

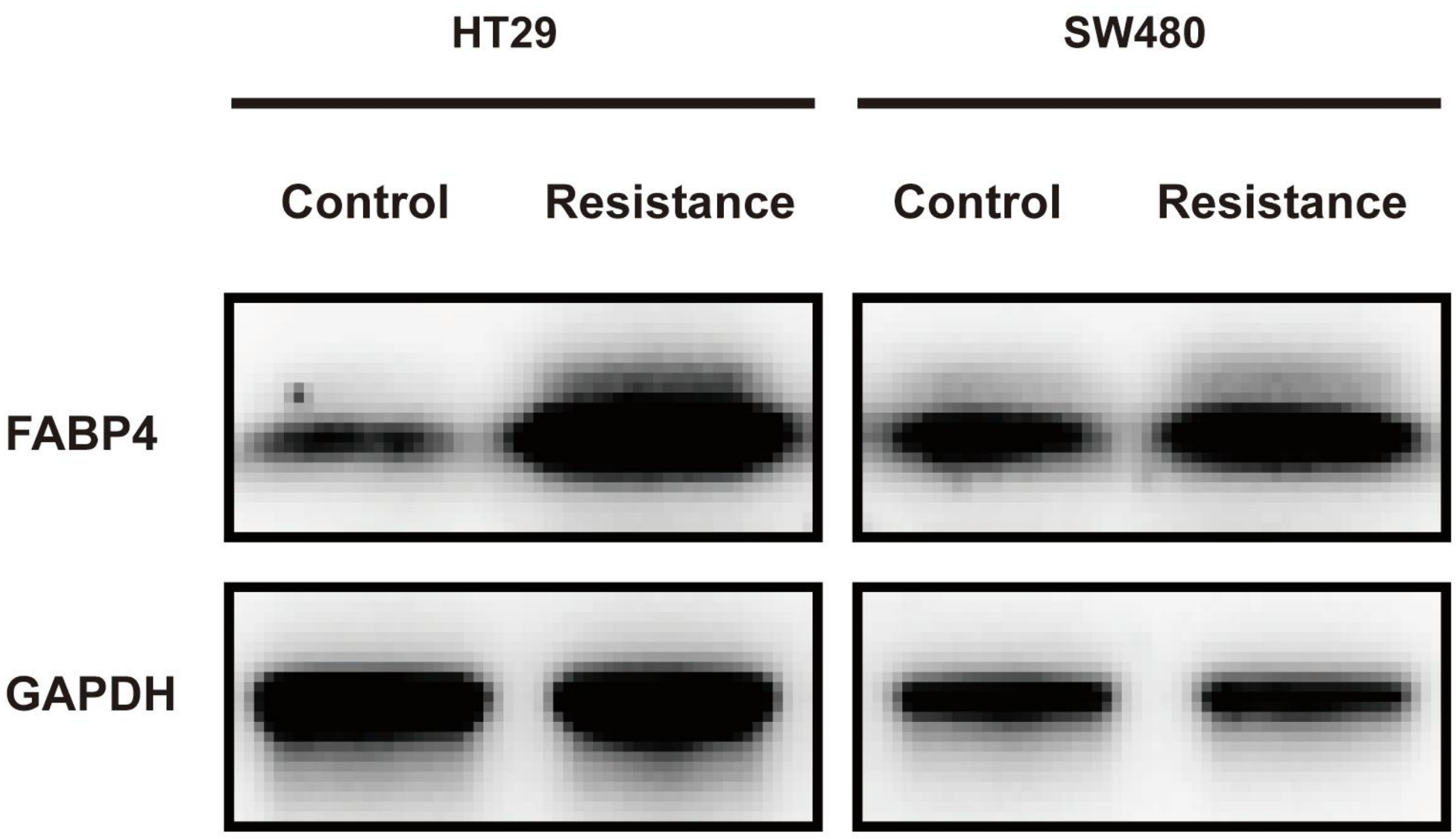

Supplementary Figure 4. The protein level of FABP4 in drug-resistant cell lines and control cells.

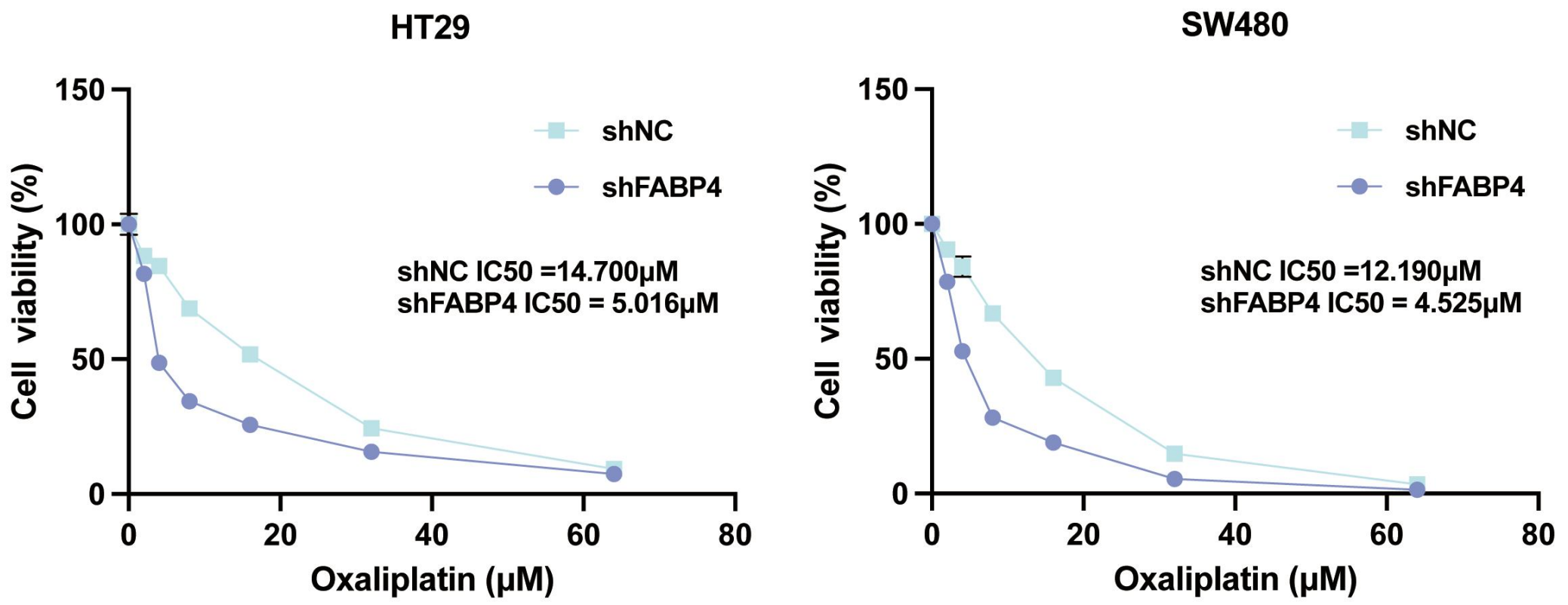

Supplementary Figure 5. FABP4 knockdown increased the sensitivity to oxaliplatin in drug-resistant cell lines.

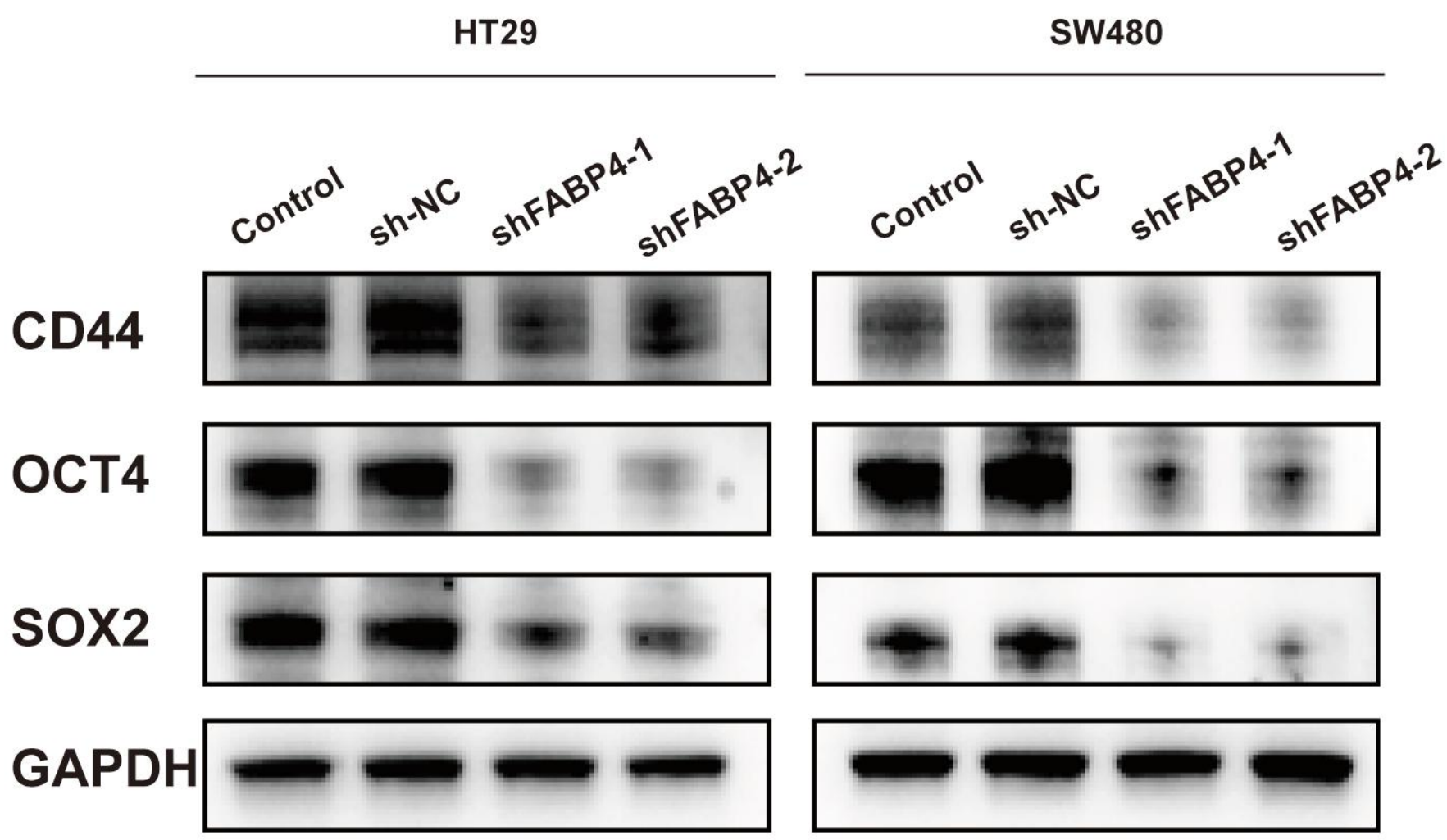

Supplementary Figure 6. Western blot analysis of stemness-related proteins (CD44, OCT4, SOX2) in drug-resistant cell lines.

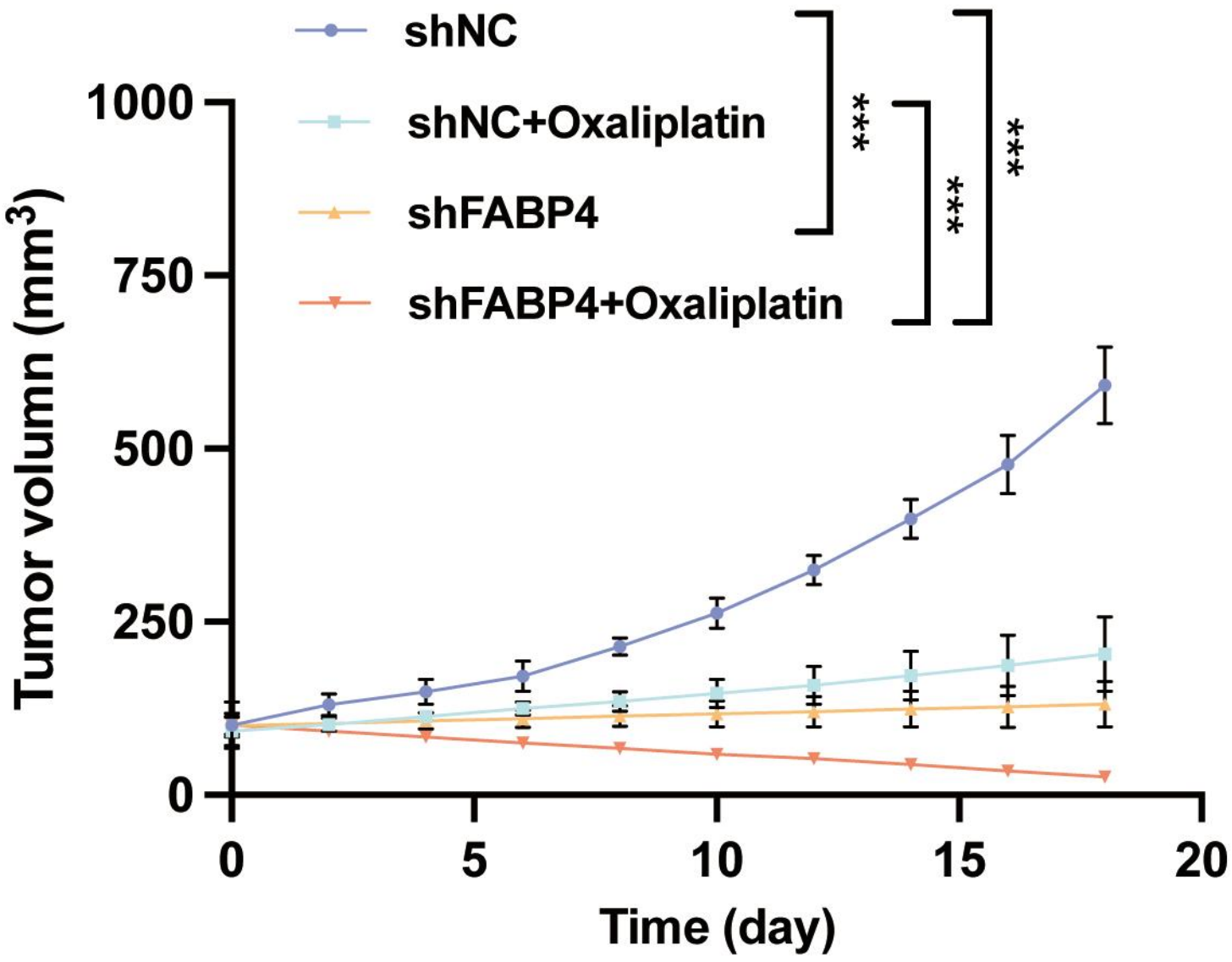

Supplementary Figure 7. Average tumor growth curves of different groups (n=5). Statistical significance was determined by two-way repeated-measures ANOVA. * indicates $p < 0.05$, ** indicates $p < 0.01$, and *** indicates $p < 0.001$.

Supplementary Table 1. IC_{50} values of oxaliplatin in HT29, SW480, and their oxaliplatin-resistant derivatives

Cell lines	IC50 (μM)
HT29	2.254
HT29/OXR	14.925
SW480	1.394
SW480/OXR	12.872

Supplementary Table 2. Primer sequences of model-related genes

Gene	Primer sequence
FABP4	F: ACTGGGCCAGGAATTTGACG
	R: CTCGTGGAAGTGACGCCTT
ADH1B	F: AGGGGGCTGTTTATGGTGG
	R: GGTACGGATACTTTTCCCAGAGT
MDFIC	F: CCACACAGGTCTGAGCAATGG
	R: AACAAGCAAGCCAGGATACAG
NOX1	F: CTGCTTCCTGTGTGTCGCAA
	R: AGGCAGATCATATAGGCCACC
GRP	F: AAAGAGCACAGGGGAGTCTTC
	R: TCCTTTGCTTCTATGAGACCCA
CCL19	F: TACATCGTGAGGAACTTCCACT
	R: CTGGATGATGCGTTCTACCCA
CALB2	F: ACTTTGACGCAGACGGAAATG
	R: GAAGTTCTCTTCGGTTGGCAG
LRP4	F: ACCTACCTGTTCCCCTCTTGA
	R: GTCCTGCTCATCCGAGTCATC

Supplementary Table 3. List of primary antibodies used in this study

**Antibodies**	**Source**	**Cat. No.**	**Lot No.**	**Dilution ratio**
FABP4	Proteintech	12802-1-AP	00160325	1:1000
CD44	Proteintech	15675-1-AP	00173417	1:1000
OCT4	Proteintech	11263-1-AP	00195078	1:1000
SOX2	Proteintech	11064-1-AP	00144863	1:1000
GAPDH	Proteintech	60004-1-Ig	00122627	1:1000
